# Feasibility of Integrating Wearable Devices and Ecological Momentary Assessment for Real-Time Environmental Exposure Estimation: Proof-of-Concept Study

**DOI:** 10.2196/86615

**Published:** 2026-05-08

**Authors:** Sameera Ramjan, Melissa Blum, Rung-Yu Tseng, Katherine Davey, Ahmed Duke Shereen, Yoko Nomura

**Affiliations:** 1 Department of Psychology, Queens College, CUNY, 65-30 Kissena Blvd, Flushing, NY, 11367, United States, 1 718-997-3164; 2 Department of Psychology, The Graduate Center, CUNY, New York, NY, United States; 3 Department of Environmental Medicine, Icahn School of Medicine at Mount Sinai, New York, NY, United States; 4 CUNY Advanced Science Research Center, New York, NY, United States; 5 Department of Psychiatry, Icahn School of Medicine at Mount Sinai, New York, NY, United States

**Keywords:** environmental exposures, exposomics, heat, air pollution, ecological momentary assessment, wearable devices, geographic information systems, mental health, mood, autonomic nervous system, mHealth, mobile health, digital health, eHealth, electronic health

## Abstract

**Background:**

Environmental exposures such as heat and air pollution are critical determinants of health, yet traditional assessment methods rely on stationary monitors or residential address proxies that fail to capture the exposures that individuals experience throughout the day.

**Objective:**

This pilot study aimed to assess the feasibility of integrating ecological momentary assessment (EMA), wearable devices, and continuous GPS tracking to capture real-time environmental exposures and to explore associations with concurrent health outcomes.

**Methods:**

In total, 7 young adults (aged approximately 16 to 24 years; 5/7, 71% female) wore Fitbit Charge 6 watches from July 2025 to August 2025 (mean 28.1, SD 1.1 days), recording sleep quality and duration, resting heart rate, breathing rate, heart rate variability, and physical activity. GPS location measured at up to 5-minute intervals (mean 19.7, SD 25.8 measurements per day) was linked to ambient temperature, humidity, and air pollution data (particulate matter <2.5 um or <10 um in diameter, nitrogen dioxide, sulfur dioxide, ozone, and carbon monoxide) derived from monitoring stations, satellites, and climate models using data-integration algorithms accessed via an application programming interface. EMA surveys administered 3 times per day captured participants’ emotional states and location (inside or outside). Feasibility targets were ≥3 GPS measurements per day, ≥1 survey completed per day, and complete sleep data on ≥50% of days. We examined exploratory bivariate correlations between environmental exposures, physiological measures, and self-reported mood, adjusting for multiple comparisons using false discovery rate correction.

**Results:**

Of the 7 participants, 5 (71%) met predefined feasibility targets. Mean compliance included 565 (SD 457) GPS coordinates per participant, 1.4 (SD 0.2) EMA surveys per day, and complete Fitbit sleep data on 64% (SD 27%) of days. Surveys identified barriers to compliance, including perceived complexity of the system and forgetting to put the Fitbit watch back on after removing it. Exploratory correlations (6/7, 86% of participants with complete Fitbit data) revealed associations between nitrogen dioxide and heat exposure and reduced heart rate variability (a marker of parasympathetic tone), and between air pollutants (sulfur dioxide) and increased negative emotions. Heat exposure showed a paradoxical pattern of lower self-reported sadness but reduced heart rate variability with higher levels of heat exposure. Given the small sample size, these correlations should be considered preliminary and hypothesis generating rather than definitive findings.

**Conclusions:**

This study demonstrates that the multimodal integration of wearable devices, GPS tracking, and EMA is feasible for capturing real-time environmental exposures and concurrent health outcomes in young adults. This approach addresses critical exposure misclassification issues in environmental health research that relies on residential addresses as proxies. Preliminary patterns suggest complex relationships between environmental exposures and both physiological and emotional outcomes, warranting further investigation in larger, more diverse samples. This approach could inform future personalized environmental health interventions.

## Introduction

Environmental exposures are well-established determinants of both physical and mental health across the life course. Prenatal exposure to heat and air pollution has been linked to altered neurodevelopment and behavior in childhood, including changes in IQ, psychomotor processing, and structural brain development [1-4]. Previous studies have also demonstrated that exposure to air pollution is associated with white matter hyperintensities and reduced functional connectivity in the frontal and parietal lobes, between the dorsal and lateral frontal cortex, and the insula [5-8]. Additionally, early exposure to ambient temperature extremes has been associated with neurodevelopmental delays, reduced myelination and maturation of white matter microstructures, and lower academic achievement [1,9,10]. These exposures also disrupt physiological processes such as sleep and autonomic nervous system activity, with implications for brain health and development [11-13].

Research in this area has been limited by imprecise exposure assessment methods and a failure to account for concurrent exposures. Most studies rely on residential or census-level data that do not capture individual movement between geographic areas of high and low exposure, particularly across indoor and outdoor environments [14,15]. Failing to account for mobility in estimating air pollution exposure has been shown to bias conclusions toward the null hypothesis in epidemiological studies [16]. This exposure misclassification limits our ability to identify which specific cognitive and behavioral domains are most affected by pollution and heat [15,17], underscoring the need for improved measurement tools that can clarify these effects and inform targeted interventions [18].

Wearable devices offer a promising solution for overcoming these limitations by combining continuous physiological monitoring with smartphone-based GPS tracking apps to record movement patterns and locations, enabling the estimation of personal exposure to temperature, air pollution, and other environmental factors in near real time [19]. When paired with ecological momentary assessment (EMA), this integrated approach can capture concurrent fluctuations in mood and behavior throughout the day, addressing key gaps in prior exposure research. This pilot study evaluated the feasibility and acceptability of integrating wearable devices, smartphone-based GPS tracking, and EMA surveys to measure individual-level heat and air pollution exposures and to explore their associations with concurrent physical and mental health outcomes (Multimedia Appendix 1). By establishing this integrated data collection framework, we aimed to lay the groundwork for scalable, real-time environmental exposure assessment in larger cohorts.

## Methods

### Study Population and Device Integration

Seven participants were recruited from Queens College, City University of New York. Participants were asked to wear a Fitbit Charge 6 (Google) continuously for 4 weeks from July 2025 to August 2025, except while charging or bathing, and were provided with written setup instructions and technical support during enrollment [20]. Data were collected via the ExpiWell (Expimetrics Inc) platform [21], a mobile app compatible with iOS and Android devices that collects health metrics from Fitbit, captures smartphone GPS data, and allows the administration of EMA surveys.

### Ethical Considerations

This protocol was approved by the institutional review board at Queens College, City University of New York (2025-0132-QC). All participants provided written informed consent. Assent and parental consent were obtained for participants aged <18 years. The privacy and confidentiality of research participants’ data were maintained. Participants did not receive compensation for participating in the study.

### Continuous GPS Tracking and Environmental Exposures

GPS tracking was performed through participants’ mobile devices at 5- to 15-minute intervals. Hourly meteorological and air pollution data for each GPS time stamp were retrieved from the OpenWeatherMap (OpenWeather Ltd) application programming interface (API), which integrates data from weather stations, satellites, and global climate models [22]. Measures included <2.5 μm and <10 μm particulate matter, ozone, nitrogen dioxide, sulfur dioxide, carbon monoxide, temperature, humidity, and heat index calculated via Rothfusz regression [23]. Environmental exposures were estimated for all GPS time points collected, regardless of participants’ reported indoor and outdoor status on EMA surveys. To preserve spatial resolution while reducing computational load, GPS coordinates were spatially binned to a 0.005° latitude or longitude grid (approximately 556 m; Figure S1 in Multimedia Appendix 2). Daily time-weighted average exposures were calculated for each participant (Multimedia Appendix 2). For a full list of available API options, see Table 1.

**Table 1.** Summary of free application programming interfaces (APIs) for air pollution data, including information on data sources, pricing, spatial and temporal resolution, and API rate limits, updated at the time of manuscript preparation.

APIs	Data sources	Price	Spatial resolution	Temporal resolution	Call limits
OpenWeatherMap [22]	SILAM^a^	Free (6-month student trial)	0.25°	Hourly	Limit of 50,000 historical calls per day; 3000 calls per minute
Open-Meteo [24]	CAMS^b^ global greenhouse gas forecast; CAMS global atmospheric composition forecast	Free	0.1°-0.25°	Hourly	Limit of 10,000 calls per day; 5000 calls per hour; 600 calls per minute
EPA^c^ AQS^d^ [25]	EPA monitoring sites nationally	Free	<25 sites in the NYC^e^ metropolitan area per time point	Hourly	Not documented
WeatherBit [26]	SILAM+CAMS+local stations	Free (21-day trial); then US $475 per month	1-15 km	Hourly	Counts each 120-hour period as a call; limit of 1500 calls per day
Open Air Quality [27]	EPA monitoring sites+local or personal publicly available sites	Free	<25 sites over NYC metropolitan area, although more than EPA AQS	Hourly	Limit of 60 calls per minute; 2000 calls per hour
Google Air Quality API [28]	Machine learning model using satellites and weather stations	Free (90-day trial with up to US $300 credit); then US $5 per 1000 calls	500 m	Hourly (up to 30 days prior)	Counts each 168-hour period as 1 call; limit of 6000 calls per minute

a SILAM: System for Integrated Modeling of Atmospheric Composition.

b CAMS: Copernicus Atmosphere Monitoring Service.

c EPA: Environmental Protection Agency.

d AQS: Air Quality System.

e NYC: New York City.

### EMA Survey

EMA surveys were administered 3 times daily (9 AM, 3 PM, and 8 PM) with 30-minute completion windows. Each survey assessed 7 emotional states (anxious, worried, nervous, sad, tired, hopeless, and happy) on a 7-point Likert scale (1=not at all to 7=extremely), as well as the participant’s location (indoors or outdoors).

### Fitbit Health Data

Fitbit metrics included daily sleep duration and stages (light, deep, and rapid eye movement); resting heart rate; heart rate variability (HRV); breathing rate; and minutes spent in active heart rate zones (fat burn, cardio, and peak). Breathing rate and HRV were available when participants wore the device during ≥3 hours of continuous sleep. Sleep quality was summarized using a composite sleep score based on duration and depth (Multimedia Appendix 2).

### Feasibility Analysis

Feasibility was evaluated by three predefined criteria: (1) at least 3 GPS measurements per participant per day with corresponding heat and pollution data, (2) at least 1 completed EMA survey per day per participant, and (3) at least 50% of days with complete Fitbit sleep and HRV data.

### Feasibility and Acceptability Surveys

After the conclusion of the 4-week pilot study, participants were invited to complete feasibility and acceptability surveys to assess their subjective experiences during the study. Participants completed Qualtrics (Qualtrics Inc) surveys evaluating the ease of using the Fitbit and ExpiWell systems (5-point Likert scales) and their comfort, enjoyment, and willingness to continue use (binary responses) [29]. Participants also provided feedback on barriers such as forgetting to wear the device or perceived inconvenience.

### Statistical Analysis

To assess agreement between exposure estimates from original and binned GPS coordinates, linear mixed-effects models were used to estimate the pairwise difference between methods with random intercepts. EMA responses were matched to daily time-weighted environmental exposures and same-day Fitbit data. Bivariate Pearson correlations (*r*) were calculated with false discovery rate correction for multiple testing (Benjamini-Hochberg method).

Due to the small sample size, observed correlations may have been driven disproportionately by a single participant. To account for this, the influence of individual participants on the results was assessed via a sensitivity score calculated as the mean absolute difference in correlation coefficients after sequentially excluding each participant (mean |Δ*r*|), with higher values indicating greater influence of single participants on the overall correlation. Correlations with mean |Δr| >0.1 were considered unstable. Given the small sample, these analyses were primarily exploratory and hypothesis generating. Analyses were conducted in R (version 4.4.1; R Foundation for Statistical Computing).

## Results

### Study Population and Device Integration

Seven participants (aged approximately 16 to 24 years; 5 female participants) were followed for 4 weeks (Table 2). One participant was excluded from Fitbit analyses due to a technical issue with data transfer but was retained for GPS analyses and EMA.

**Table 2.** Study follow-up and evaluation of missing data.

Participant ID	GPS-EMA^a^ follow-up (days; mean 28.7, SD 1.0), n	Fitbit follow-up (days; mean 28.1, SD 1.1), n	Total GPS points (mean 565, SD 457)	Daily GPS points (mean 19.7, SD 25.8), mean (SD)	Total EMA surveys completed (mean 39.7, SD 18.1), n	Daily EMA surveys (mean 1.4, SD 0.2), mean (SD)	Percentage of days with Fitbit data (mean 71.2, SD 33.0; %)
1	29	27	1058	36.5 (38.4)	68	2.4 (0.6)	74.1
2	30	30	503	16.8 (21.9)	32	1.1 (0.8)	93.3
3	29	28	153	5.3 (5.9)	41	1.4 (1.1)	71.4
4	29	28	21	0.7 (1.1)	15	0.5 (0.7)	0.0
5	29	29	879	30.3 (40.7)	46	1.6 (0.8)	96.6
6	27	27	212	7.9 (6.0)	53	2.0 (0.9)	88.9
7	28	28	1128	40.3 (72.8)	23	0.8 (0.9)	75.0

a EMA: ecological momentary assessment.

### Feasibility Analysis

Participants recorded a mean of 19.7 (SD 25.8) GPS measurements per day, with 6 participants meeting the feasibility goal of ≥3 daily measurements (Table 2; Figure S2 in Multimedia Appendix 2). Participants completed a mean of 1.4 (SD 0.2) EMA surveys per day. Of the 7 participants, 5 (71%) met the compliance goal of ≥1 survey per day (Table 2). EMA responses are shown in Table 3. Moreover, 5 (71%) of 7 participants met the ≥50% completeness goal for sleep and sleep-associated Fitbit metrics (Table 4).

**Table 3.** Ecological momentary assessment (EMA) responses^a^.

Participant ID	Anxious	Worried	Nervous	Sad	Hopeless	Tired	Happy	Time spent indoors (%)
Emotional state scores, mean (SD)								
1	1.19 (0.40)	1.22 (0.42)	1.26 (0.51)	1.21 (0.48)	1.24 (0.52)	2.00 (1.28)	2.55 (1.43)	52.2
2	2.29 (0.81)	2.75 (1.04)	2.68 (0.95)	2.62 (1.10)	3.08 (0.74)	2.85 (1.26)	3.08 (0.74)	88.5
3	2.42 (1.22)	2.71 (1.20)	2.31 (0.85)	2.22 (0.83)	1.33 (0.52)	3.64 (1.31)	4.73 (1.23)	63.4
4	1.86 (0.77)	1.93 (0.73)	1.64 (0.74)	1.18 (0.40)	Not estimable	3.93 (0.80)	3.40 (0.63)	80.0
5	2.63 (0.68)	2.37 (0.85)	2.48 (0.82)	2.56 (0.80)	2.12 (0.60)	2.58 (1.18)	3.29 (1.36)	68.9
6	1.29 (0.47)	1.68 (0.57)	1.29 (0.49)	1.00 (0.00)	1.00 (0.00)	2.32 (1.05)	5.15 (0.82)	79.2
7	1.87 (1.69)	1.96 (1.38)	1.57 (0.89)	2.30 (1.90)	1.83 (1.39)	3.09 (2.17)	2.13 (1.20)	78.3
Overall	1.9 (0.6)	2.1 (0.6)	1.9 (0.6)	1.9 (0.7)	1.8 (0.8)	2.9 (0.7)	3.5 (1.1)	72.9 (12.2)
Missing EMA data (%)								
1	0	0	0	0	0	0	1.5	1.5
2	12.5	12.5	21.9	18.8	18.8	18.8	18.8	18.8
3	53.7	65.9	68.3	78.0	85.4	12.2	0	0
4	6.7	6.7	6.7	26.7	100	0	0	0
5	0	0	4.3	6.5	28.3	6.5	2.2	2.2
6	73.6	58.5	86.8	94.3	81.1	41.5	0	0
7	0	0	0	0	0	0	0	0
Overall	20.9	20.5	26.9	32	44.8	11.3	3.2	3.2

a Descriptive statistics and missingness are presented for all EMA surveys completed by each participant during the study period.

**Table 4.** Fitbit-derived health data^a^.

Participant ID^b^	Sleep duration^c^ (hours)	Sleep score^d^	Breathing rate (breaths per minute)	Daily HRV^e^^,^^f^	Deep sleep HRV^g^	Resting heart rate^h^ (beats per minute)	Active zone duration^i^ (minutes)
Fitbit health parameters, mean (SD)							
1	7.85 (2.16)	0.91 (0.11)	13.84 (0.50)	35.98 (4.22)	33.47 (5.52)	71.59 (2.35)	4.88 (3.52)
2	7.74 (2.22)	0.91 (0.09)	17.22 (1.21)	26.04 (6.71)	28.19 (7.75)	76.93 (1.88)	16.14 (22.79)
3	7.42 (2.24)	0.82 (0.12)	18.47 (1.19)	44.42 (5.63)	40.38 (5.99)	60.25 (1.89)	6.36 (7.10)
5	7.40 (1.63)	0.93 (0.08)	18.49 (0.72)	57.19 (15.20)	59.05 (17.99)	72.82 (3.13)	50.31 (49.31)
6	7.34 (1.52)	0.90 (0.14)	16.90 (0.84)	39.60 (8.48)	37.32 (13.94)	73.50 (3.25)	26.83 (28.35)
7	7.41 (1.85)	0.90 (0.10)	14.52 (0.93)	28.87 (3.98)	23.72 (11.12)	68.81 (4.63)	90.94 (67.33)
Overall	7.5 (0.2)	0.9 (0.0)	16.6 (2.0)	38.7 (11.3)	37.0 (12.4)	70.7 (5.7)	32.6 (33.1)
Missing data (%)							
1	25.9	25.9	29.6	29.6	29.6	37.0	70.4
2	16.7	16.7	16.7	16.7	16.7	10.0	30.0
3	38.5	38.5	42.3	38.5	38.5	23.1	57.7
5	17.2	17.2	17.2	17.2	17.2	3.4	10.3
6	88.6	88.6	88.6	88.6	88.6	77.1	34.3
7	31.0	31.0	41.4	31.0	31.0	27.6	37.9
Overall	36.3	36.3	29.7	39.3	40.1	36.9	36.9

a For all participants with any complete Fitbit data, descriptive statistics and missingness are presented for all Fitbit-derived health parameters collected during the study period.

b Participant 4 skipped this survey item on all surveys and an average cannot be calculated.

c Sleep duration refers to total hours spent sleeping within a 24-hour period, including naps.

d Sleep score represents sleep quality on a scale from 0 to 100 (optimal), based on sleep duration and time spent in rapid eye movement and deep sleep phases.

e HRV: heart rate variability.

f Daily HRV refers to the average daily root mean square of successive differences of heart rate.

g Deep sleep HRV refers to root mean square of successive differences of heart rate during deep sleep phases from the longest sleep period in the past day.

h Resting heart rate refers to average daily heart rate while the participant is still and well rested.

i Active zone duration refers to daily minutes with heart rate elevated above resting levels.

### Feasibility and Acceptability Surveys

Compliance data identified several feasibility challenges. One participant experienced a technical issue with Fitbit data transfer, and another showed moderate to high missingness in sleep and sleep-related metrics. Among the 4 participants who completed feasibility questionnaires (Table S3 in Multimedia Appendix 2), 3 reported forgetting to put the Fitbit device back on after removing it and 2 cited perceived annoyance and reluctance to wear the Fitbit for longer periods, consistent with observed gaps in sleep data.

Compliance was further limited by a low EMA response rate in 2 participants and greater missingness on survey items assessing negative emotions, which could introduce bias. One participant with a low EMA response rate also demonstrated poor GPS data compliance. On feasibility surveys, 1 (25%) of 4 participants perceived the system as overly complex and difficult to use without technical support, and 2 participants reported that there was a lot to learn before they could begin using it. These responses may reflect difficulty navigating the survey interface and remembering to keep the ExpiWell app open to allow for continuous GPS tracking.

### Environmental Exposure Estimation

**Figure 1.**
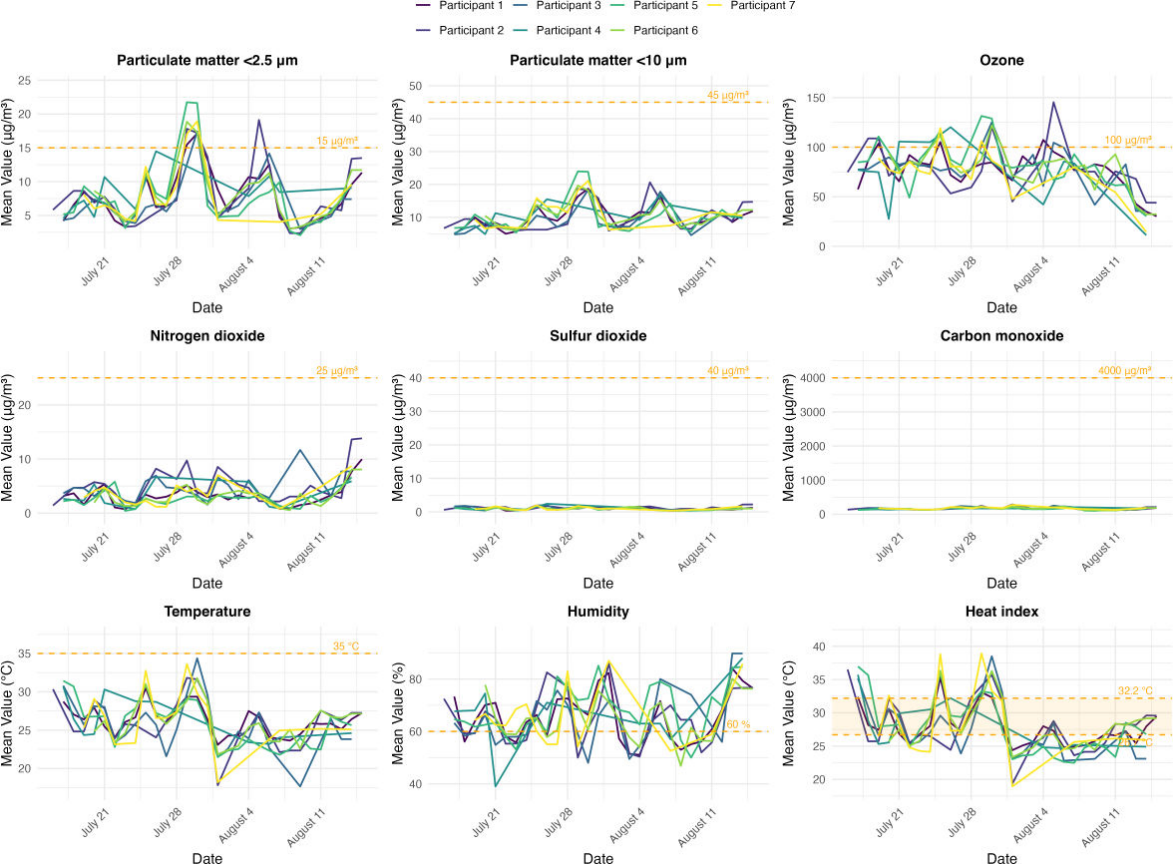
Daily time-weighted average environmental exposures are shown for each participant over the study period, with safe exposure limits indicated by dashed lines. For air pollutants, 24-hour safe exposure limits based on World Health Organization guidelines are shown, except for ozone, for which the 8-hour safe limit is shown. For temperature and humidity, safe limits are shown based on the literature assessing the risk of heat-related morbidity and discomfort. Heat index was derived from Rothfusz regression (°C). Aug: August; Jul: July.

Across all pollutants and meteorological variables examined, environmental exposures extracted with and without coordinate binning were highly correlated (Pearson *r* >0.99), and the estimated mean differences between exposures were not statistically significant (Table S1 in Multimedia Appendix 2). Daily time-weighted average exposures for each participant are shown in Figure 1 [30-33] and Table S2 in Multimedia Appendix 2. Mean environmental exposure estimates varied by indoor and outdoor status throughout the study period (Figure S3 in Multimedia Appendix 2).

### Exploratory Associations Between Environmental Exposures and Health Indicators

**Figure 2.**
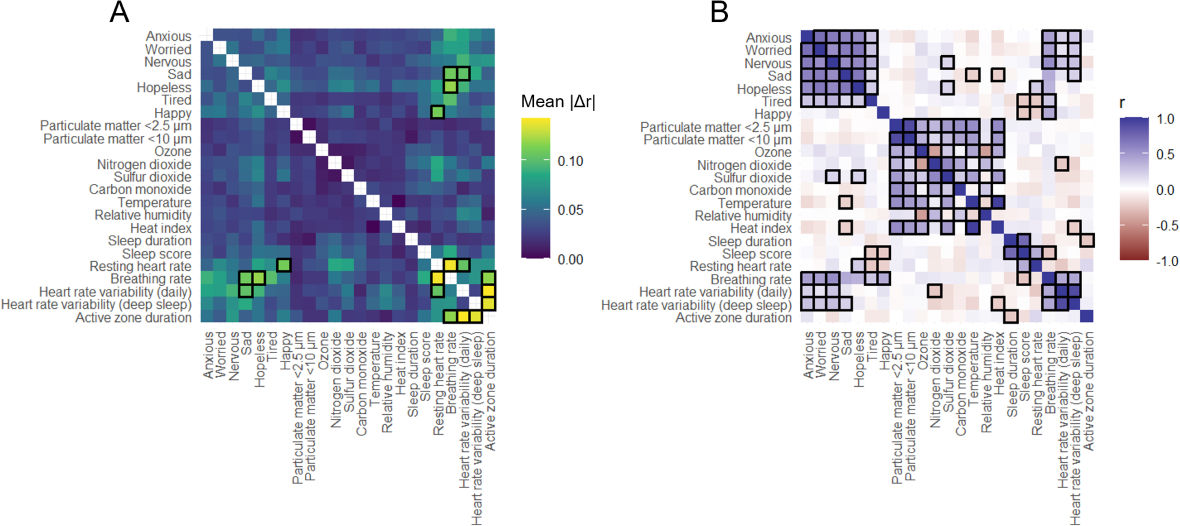
Bivariate correlations and sensitivity analysis of environmental exposures, Fitbit-derived health parameters, and emotional state. (A) A heat map of sensitivity indices (mean ΙΔ*r*Ι) is shown for each bivariate correlation between continuous variables (correlations with mean ΙΔ*r* >0.1 are outlined in black, indicating an unstable correlation), and (B) a heat map of Pearson correlation coefficients is shown for all bivariate correlations between continuous variables (correlations with an adjusted *P* value <.05 and mean ΙΔ*r*Ι <0.1 are outlined in black). Heat index: index derived from Rothfusz regression (°C); sleep duration: total hours spent sleeping within a 24-hour period, including naps; sleep score: representation of sleep quality on a scale from 0 to 100 (optimal) based on sleep duration and time spent in rapid eye movement and deep sleep phases; heart rate variability (daily): average daily root mean square of successive differences of heart rate; heart rate variability (deep sleep): root mean square of successive differences of heart rate during deep sleep phases from the longest sleep period over the past day; resting heart rate: average daily heart rate while participant was still and well rested; breathing rate: average daily breaths per minute; active zone duration: daily minutes with heart rate elevated above resting.

Bivariate correlations indicated potential relationships between environmental exposures and physiologic or emotional measures; however, directionality and causation cannot be inferred. Most correlations were stable to the exclusion of single participants (sensitivity index <0.1; Figure 2). Unstable correlations were excluded from interpretation due to the potential for single participants to bias the results in a small sample. Higher heat index (*r*=−0.22; *P*=.02) and nitrogen dioxide (*r*=−0.24; *P*=.01) levels were associated with lower HRV, suggesting reduced parasympathetic tone. Sulfur dioxide exposure correlated with higher “nervous” (*r*=0.19; *P*=.04) and “hopeless” (*r*=0.21; *P*=.04) scores. Conversely, higher heat index exposure was associated with lower “sad” (*r*=−0.21; *P*=.03) scores. See correlation coefficients, *P* values, and sensitivity indices in Tables S4 to S6 in Multimedia Appendix 2.

## Discussion

This proof-of-concept study demonstrates the feasibility of integrating wearable devices, smartphone-based GPS tracking, and EMA to capture real-time environmental exposures and behavioral fluctuations. Although wearable devices are already being used to study the health impacts of climate change [34,35], our framework successfully combined physiological, geospatial, and mood data from commercially available tools, producing synchronized multimodal datasets suitable for scalable environmental health research.

Open-access environmental data sources such as the OpenWeatherMap API offer cost-effective near real-time exposure estimates without specialized monitoring equipment. Validation of our GPS spatial binning approach confirmed that simplified data processing can maintain high spatial accuracy, supporting its use in larger cohorts. Despite the small sample, our methodology helps address exposure misclassification, a major limitation of prior studies relying on residential addresses for interpolation without indoor or outdoor contextualization [36]. User feedback suggested that this novel approach is both technically feasible and acceptable to participants, while identifying additional opportunities to improve usability and compliance.

Preliminary analyses suggested patterns between environmental exposures and both emotional and physiological indicators. Higher sulfur dioxide exposure was associated with greater negative emotionality, consistent with prior evidence linking air pollution to psychiatric symptoms such as anxiety and depression [37]. Although previous studies have linked heat exposure to increased mental health–related emergency department visits [38], the relationship between heat and mood in our small sample was less clear, with some suggestion of lower sadness scores during periods of higher heat exposure; however, this may reflect nonlinear associations, unmeasured confounding, or the small sample size. Both heat pollution and air pollution were negatively correlated with HRV, consistent with previous reports of increased sympathetic activation and reduced parasympathetic tone [39-41]. Given the small sample size, these findings should be interpreted primarily as exploratory and hypothesis generating and are not intended to be generalizable.

Several limitations warrant consideration. Technical issues and participant noncompliance led to missing Fitbit and GPS tracking data, which may have biased results if data loss was related to activity patterns or time of day. Exposure estimates were based on modeled outdoor environmental data rather than direct sensor measurements, and indoor-outdoor differences were not measured. The small convenience sample of research assistants from a single university limits generalizability. The response rate to the feasibility and acceptability surveys was low, and participants who experienced greater difficulties may not have returned the survey, limiting the interpretation of positive responses. Finally, no incentive was provided to participants, which may have affected study compliance.

Despite these limitations, this framework holds promise for studying the acute and cumulative effects of environmental stressors. Although larger, more diverse cohorts are needed to validate and extend these results, this proof-of-concept study demonstrates a scalable and participant-acceptable framework for capturing the real-time impacts of environmental exposures. Ultimately, such approaches may help clarify mechanisms linking environmental stressors to health outcomes, inform public health interventions, and guide personalized approaches to risk reduction.

## Supplementary material

10.2196/86615 Multimedia Appendix 1 Graphical abstract.

10.2196/86615 Multimedia Appendix 2 Supplementary equations, figures, and tables detailing data processing methods, environmental exposure estimation, and additional analyses.

## Abbreviations

API: application programming interface
EMA: ecological momentary assessment
HRV: heart rate variability

## References

[1] Lin Q, Gao Y, Liu Y (2025). Heat wave exposure during pregnancy and neurodevelopmental delay in young children: a birth cohort study. Environ Res.

[2] DeIngeniis D, Blum M, Lee RM, Shereen AD, Nomura Y (2025). Prenatal exposure to extreme ambient heat may amplify the adverse impact of Superstorm Sandy on basal ganglia volume among school-aged children. PLoS One.

[3] Guxens M, Garcia-Esteban R, Giorgis-Allemand L (2014). Air pollution during pregnancy and childhood cognitive and psychomotor development: six European birth cohorts. Epidemiology.

[4] Kim E, Park H, Hong YC (2014). Prenatal exposure to PM₁₀ and NO₂ and children’s neurodevelopment from birth to 24 months of age: Mothers and Children’s Environmental Health (MOCEH) study. Sci Total Environ.

[5] Herting MM, Younan D, Campbell CE, Chen JC (2019). Outdoor air pollution and brain structure and function from across childhood to young adulthood: a methodological review of brain MRI studies. Front Public Health.

[6] Calderón-Garcidueñas L, Engle R, Mora-Tiscareño A (2011). Exposure to severe urban air pollution influences cognitive outcomes, brain volume and systemic inflammation in clinically healthy children. Brain Cogn.

[7] Pujol J, Martínez-Vilavella G, Macià D (2016). Traffic pollution exposure is associated with altered brain connectivity in school children. Neuroimage.

[8] Cotter DL, Campbell CE, Sukumaran K (2023). Effects of ambient fine particulates, nitrogen dioxide, and ozone on maturation of functional brain networks across early adolescence. Environ Int.

[9] Granés L, Essers E, Ballester J (2024). Early life cold and heat exposure impacts white matter development in children. Nat Clim Chang.

[10] Kidd SA, Gong J, Massazza A, Bezgrebelna M, Zhang Y, Hajat S (2023). Climate change and its implications for developing brains – in utero to youth: a scoping review. J Clim Chang Health.

[11] Chevance G, Minor K, Vielma C (2024). A systematic review of ambient heat and sleep in a warming climate. Sleep Med Rev.

[12] Taylor-Clark TE (2020). Air pollution-induced autonomic modulation. Physiology (Bethesda).

[13] Berger SE, Ordway MR, Schoneveld E (2023). The impact of extreme summer temperatures in the United Kingdom on infant sleep: implications for learning and development. Sci Rep.

[14] Shang L, Yang L, Yang W (2020). Effects of prenatal exposure to NO_2_ on children’s neurodevelopment: a systematic review and meta-analysis. Environ Sci Pollut Res Int.

[15] Thompson R, Smith RB, Karim YB (2023). Air pollution and human cognition: a systematic review and meta-analysis. Sci Total Environ.

[16] Setton E, Marshall JD, Brauer M (2011). The impact of daily mobility on exposure to traffic-related air pollution and health effect estimates. J Expo Sci Environ Epidemiol.

[17] Fischer S, Naegeli K, Cardone D (2024). Emerging effects of temperature on human cognition, affect, and behaviour. Biol Psychol.

[18] Pratt GC, Vadali ML, Kvale DL, Ellickson KM (2015). Traffic, air pollution, minority and socio-economic status: addressing inequities in exposure and risk. Int J Environ Res Public Health.

[19] Webber SC, Porter MM (2009). Monitoring mobility in older adults using global positioning system (GPS) watches and accelerometers: a feasibility study. J Aging Phys Act.

[20] Fitbit Charge 6 tracker. Google.

[21] ExpiWell.

[22] Accuracy and quality of weather data. OpenWeather.

[23] Rothfusz LP The Heat Index “equation” (or, more than you ever wanted to know about Heat Index). National Weather Service.

[24] Free weather API. Open-Meteo.

[25] Air Quality System (AQS) API. United States Environmental Protection Agency.

[26] Weather API. Weatherbit.

[27] OpenAQ.

[28] Air quality API overview. Google.

[29] Qualtrics.

[30] (2021). WHO global air quality guidelines: particulate matter (PM2.5 and PM10), ozone, nitrogen dioxide, sulfur dioxide and carbon monoxide. World Health Organization.

[31] Arundel AV, Sterling EM, Biggin JH, Sterling TD (1986). Indirect health effects of relative humidity in indoor environments. Environ Health Perspect.

[32] What is the heat index?. National Weather Service.

[33] McGregor GR, Bessemoulin P, Ebi K, Menne B (2015). Heatwaves and health: guidance on warning-system development. World Meteorological Organization and World Health Organization.

[34] Dolson CM, Harlow ER, Phelan DM (2022). Wearable sensor technology to predict core body temperature: a systematic review. Sensors (Basel).

[35] Zhou P, Ma J, Li X (2023). The long-term and short-term effects of ambient air pollutants on sleep characteristics in the Chinese population: big data analysis from real world by sleep records of consumer wearable devices. BMC Med.

[36] Kazakos V, Luo Z, Ewart I (2020). Quantifying the health burden misclassification from the use of different PM_2.5_ exposure tier models: a case study of London. Int J Environ Res Public Health.

[37] Radua J, De Prisco M, Oliva V, Fico G, Vieta E, Fusar-Poli P (2024). Impact of air pollution and climate change on mental health outcomes: an umbrella review of global evidence. World Psychiatry.

[38] Niu L, Girma B, Liu B, Schinasi LH, Clougherty JE, Sheffield P (2023). Temperature and mental health-related emergency department and hospital encounters among children, adolescents and young adults. Epidemiol Psychiatr Sci.

[39] Brenner IK, Thomas S, Shephard RJ (1997). Spectral analysis of heart rate variability during heat exposure and repeated exercise. Eur J Appl Physiol Occup Physiol.

[40] Baja ES, Schwartz JD, Coull BA, Wellenius GA, Vokonas PS, Suh HH (2013). Structural equation modeling of parasympathetic and sympathetic response to traffic air pollution in a repeated measures study. Environ Health.

[41] Chen CH, Lai F, Huang LY, Guo YLL (2024). Short- and medium-term cumulative effects of traffic-related air pollution on resting heart rate in the elderly: a wearable device study. Ecotoxicol Environ Saf.

