# Supplementary figures and images for: Feasibility of Integrating Wearable Devices and Ecological Momentary Assessment for Real-Time Environmental Exposure Estimation: Proof-of-Concept Study
Source: JMIR Form Res. 2026 May 8;10:e86615. doi: 10.2196/86615 (PMC13155499; doi:10.2196/86615)

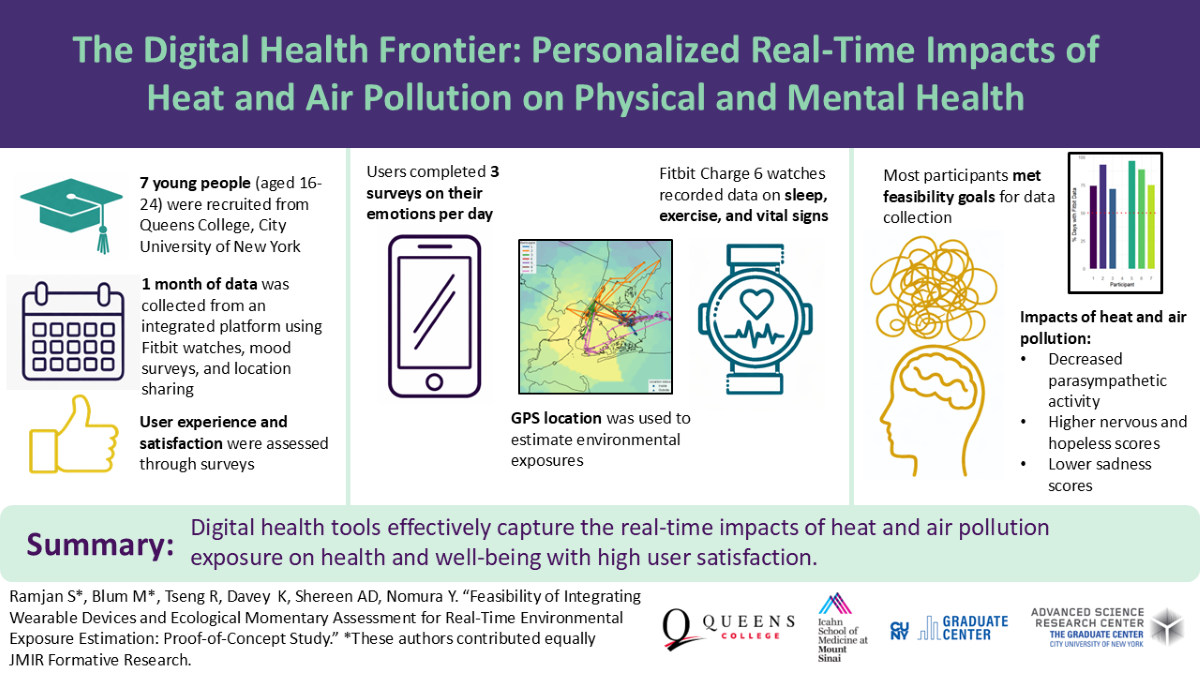

Supplement: Multimedia Appendix 1 [file formative-v10-e86615-s001.png]
