# Supplementary material for: Feasibility of Integrating Wearable Devices and Ecological Momentary Assessment for Real-Time Environmental Exposure Estimation: Proof-of-Concept Study
Source: JMIR Form Res. 2026 May 8;10:e86615. doi: 10.2196/86615 (PMC13155499; doi:10.2196/86615)
Supplement: Multimedia Appendix 2 [file formative-v10-e86615-s002.docx]

**Supplementary Material**

**Daily Time-Weighted Average Environmental Exposure Calculation**

Daily time-weighted average exposures were calculated as the sum of each exposure estimate multiplied by the time interval to the next timestamp (capped at 15 minutes), divided by the total accumulated (capped) time over the day, defined as the 24-hour period ranging from midnight to midnight in the participant’s local time zone.

$$Daily_{twavg}= \frac{\sum_{n=1}^{N-1} E_{n}*{\Delta t}_{n}}{\sum_{n=1}^{N-1} {\Delta t}_{n}}$$

Where:

- $E_{n}$ = exposure estimate at timestamp n
- Δ$t_{n}$ = min($t_{n+1}- t_{n}$, 15 minutes)
- $t_{n}$ = timestamp n
- $N$ = total number of unique GPS timestamps in that 24-hour period

**Sleep Score Calculation**

Sleep parameters were derived from Fitbit output. Missing values were imputed as zero minutes for each sleep stage. Sleep scores were calculated only for days with at least 1 sleep period recorded. The following variables were calculated:

- **Total sleep duration (hours):** Sum of minutes spent in each sleep stage for long (*deep, light, REM,* and *wake)* and short *(awake, restless,* and *asleep)* sleep periods divided by 60.
- **Duration score:** Proportional to recommended sleep duration of 7.5 hours, defined as

Duration Score = {1.0 if total sleep duration ≥ 7.5; otherwise, total sleep duration/7.5}

- **Long sleep depth score:** Ratio of *deep + REM* sleep to total minutes in *deep + REM + light + wake* sleep stages, proportional to the recommended fraction of 0.4.

Long Sleep Depth Score = {1.0 if Deep + REM / (Deep + REM + Light + Wake) ≥ 0.4; otherwise, (Deep + REM / (Deep + REM + Light + Wake))/0.4}

- **Short sleep depth score:** Ratio of *asleep minutes* to total minutes in *asleep + restless + awake* sleep stages.

Short Sleep Depth Score = Asleep / (Asleep + Restless + Awake)

- **Overall depth score:** Average of short and long sleep depth scores (ignoring missing values)
- **Composite sleep score:** Weighted average of duration and depth scores, where:

Sleep Score = 2/3 * Duration Score + 1/3 * Overall Depth Score

All metrics were calculated at the participant-day level.

**Supplementary Figure 1. Map of original and spatially binned GPS coordinates**


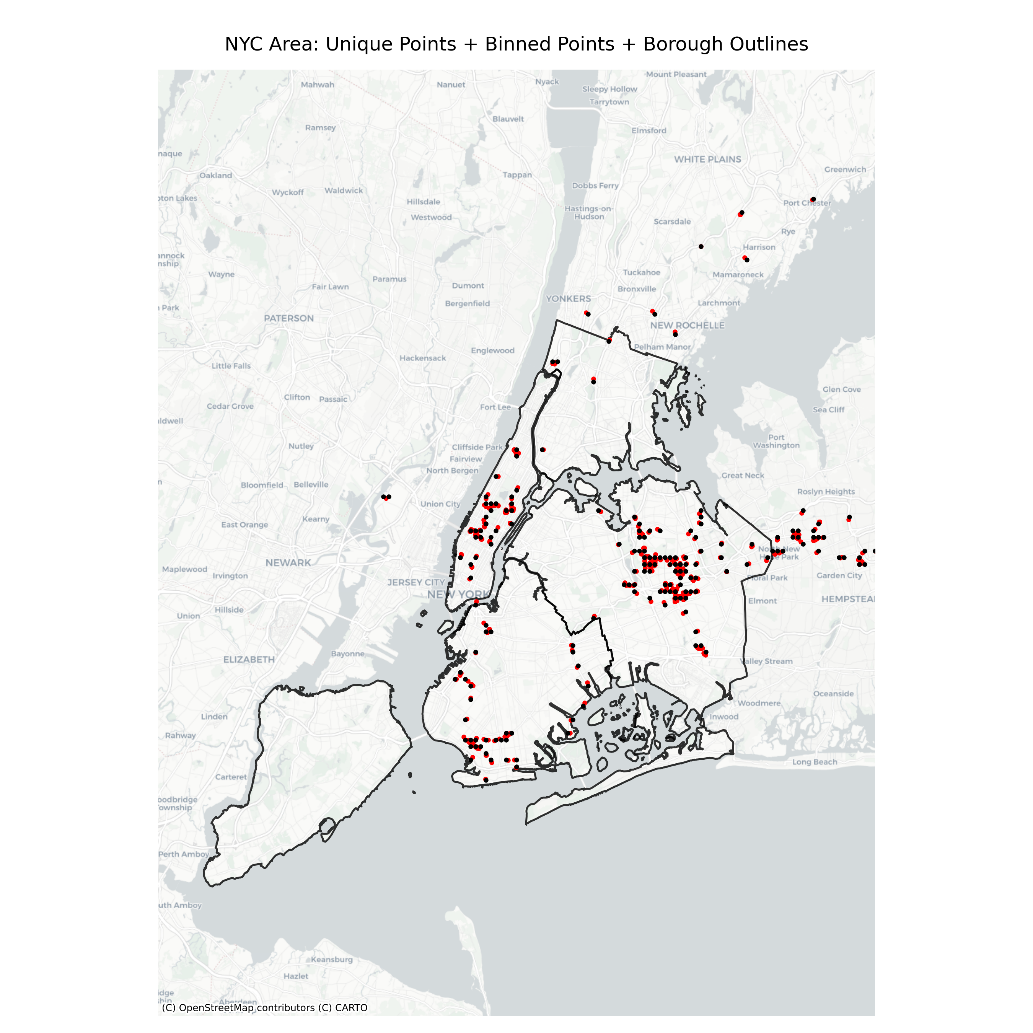


All unique GPS coordinates collected in the New York City Metro Area are shown as red points superimposed on a map of the five boroughs. Black points represent the same coordinates binned to a 0.005° grid.

**Supplementary Figure 2. Interpolating Environmental Exposures from GPS Tracking Data**


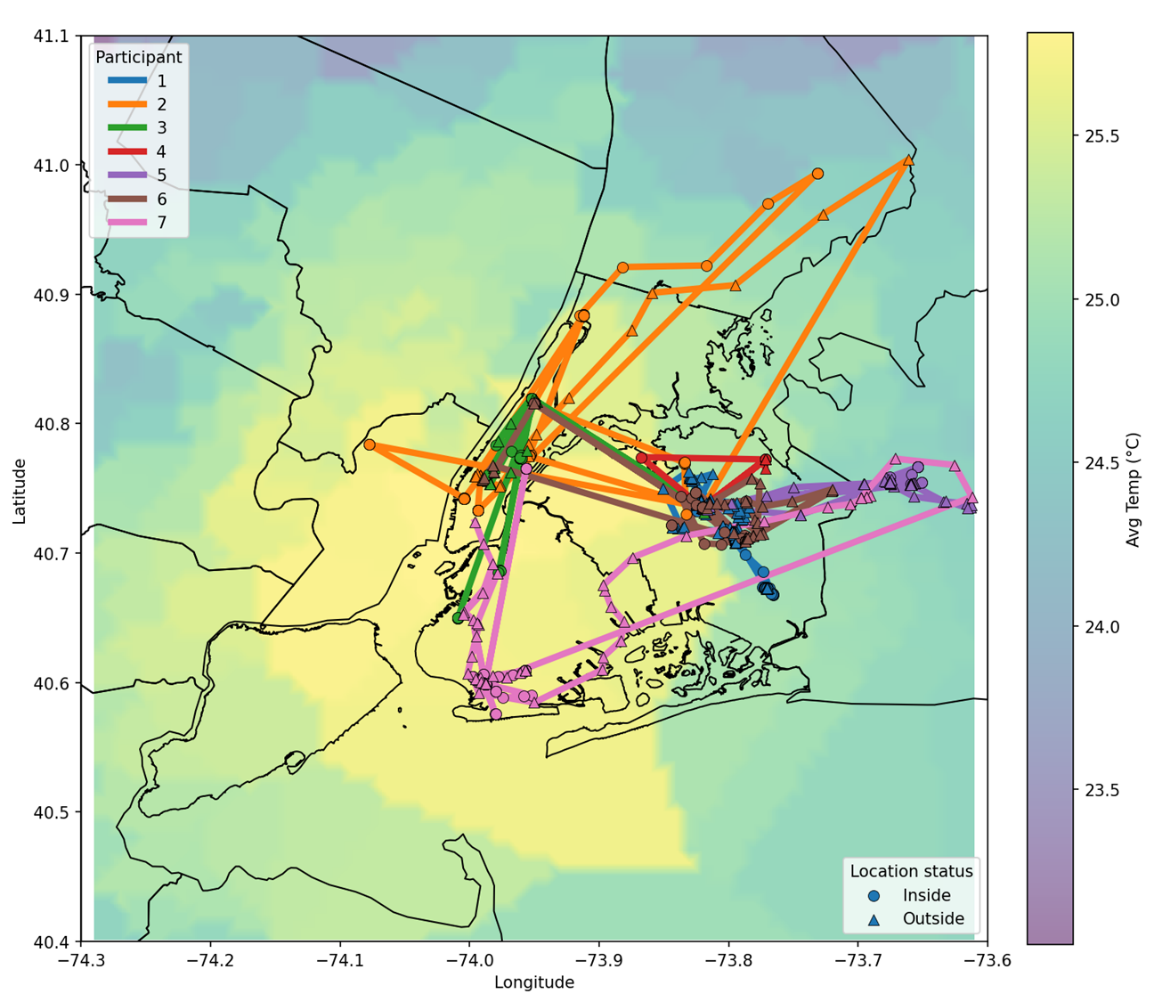


A heat map of average temperature during the 4-week study period is shown for the New York City metro area. Participant GPS locations are indicated by circles if the closest survey completed indicated their location was indoors, and triangles if the closest survey indicated they were outdoors. Points are color-coded by participant. Avg temp = average ambient temperature (°C) over the 4-week study period.

**Supplementary Figure 3. Environmental Exposures by Indoor/Outdoor Status**


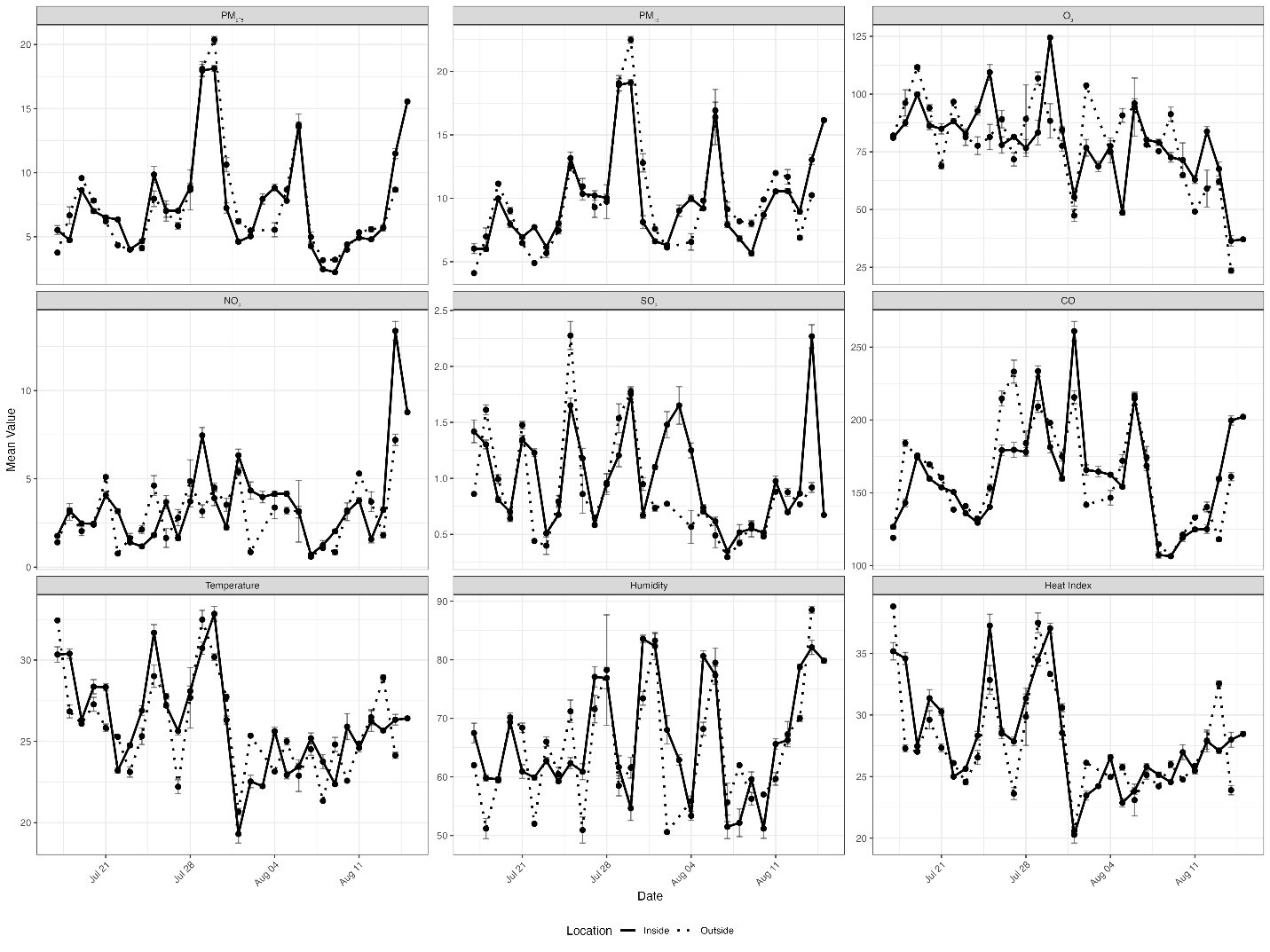


Mean environmental exposure estimates are shown for each day throughout the study period, stratified by whether the participant indicated they were indoors or outdoors on the closest survey within 3 hours of the GPS timestamp from which exposure estimates were derived. A solid line indicates that the participant was indoors, while a dotted line indicates they were outdoors. PM_2.5_ = particulate matter < 2.5 μm (μg/m^3^); PM_10_ = particulate matter < 10 μm (μg/m^3^); CO = carbon monoxide (μg/m^3^); NO_2_ = nitrogen dioxide (μg/m^3^); SO_2_ = sulfur dioxide (μg/m^3^); O_3_ = ozone (μg/m^3^); °C = degrees Celsius; Temperature = ambient temperature (°C); Humidity = relative humidity (%); Heat index = heat index derived from Rothfusz regression (°C).**Supplementary Table 1. Mixed Effects Comparison of Exposure Estimates from Original and Binned Coordinates**

| **Exposure** | **Intercept (95% CI)** | ***P-value*** | **Pearson’s *r*** | **MAE** |
| --- | --- | --- | --- | --- |
| PM_2.5_ (μg/m^3^) | 0.005 (-0.009, 0.018) | .40 | 1.000 | 0.004 |
| PM_10_ (μg/m^3^) | 0.005 (-0.010, 0.021) | .40 | 0.999 | 0.004 |
| CO (μg/m^3^) | 0.017 (-0.031, 0.065) | .38 | 1.000 | 0.018 |
| NO_2_ (μg/m^3^) | -0.001 (-0.003, 0.001) | .36 | 1.000 | 0.001 |
| SO_2_ (μg/m^3^) | 0.000 (-0.002, 0.001) | .38 | 1.000 | 0.000 |
| O_3_ (μg/m^3^) | -0.006 (-0.024, 0.013) | .44 | 1.000 | 0.010 |
| Temperature (°C) | -0.002 (-0.007, 0.004) | .56 | 1.000 | 0.007 |
| Heat Index (°C) | 0.000 (-0.009, 0.008) | .93 | 1.000 | 0.011 |

For each environmental exposure, the fixed intercept and *P*-value representing the estimated overall mean difference between exposures extracted from original vs binned GPS coordinates, adjusted for within-participant and within-participant-day correlations, is shown. Pearson correlation coefficients were calculated between exposure estimates from original vs binned GPS coordinates. Mean absolute error (MAE) represents the average absolute difference between estimates generated from original vs. binned coordinates. 95% CI = 95% confidence interval; PM_2.5_ = particulate matter < 2.5 μm (μg/m^3^); PM_10_ = particulate matter < 10 μm (μg/m^3^); CO = carbon monoxide (μg/m^3^); NO_2_ = nitrogen dioxide (μg/m^3^); SO_2_ = sulfur dioxide (μg/m^3^); O_3_ = ozone (μg/m^3^); Temperature = ambient temperature (°C); Heat Index = heat index derived from Rothfusz regression (°C).

**Supplementary Table 2. Average Daily Time-Weighted Environmental Exposures by Participant**

| **Participant ID** | **Environmental Exposures (Daily Time-Weighted Mean (SD))** | | | | | | | | |
| --- | --- | --- | --- | --- | --- | --- | --- | --- | --- |
|  | **PM_2.5_ (μg/m^3^)** | **PM_10_ (μg/m^3^)** | **O_3_ (μg/m^3^)** | **NO_2_ (μg/m^3^)** | **SO_2_ (μg/m^3^)** | **CO (μg/m^3^)** | **Temperature (**°**C)** | **Humidity (%)** | **Heat Index (**°**C)** |
| 1 | 8.69 (3.60) | 10.26 (3.51) | 72.75 (22.49) | 3.93 (2.39) | 1.09 (0.49) | 164.57 (25.31) | 26.22 (1.90) | 68.54 (11.40) | 27.64 (2.63) |
| 2 | 8.44 (4.27) | 10.78 (4.06) | 73.40 (24.12) | 5.74 (3.84) | 1.42 (0.50) | 162.84 (30.71) | 26.66 (2.51) | 66.88 (11.07) | 28.45 (3.61) |
| 3 | 6.00 (2.53) | 7.98 (2.84) | 78.05 (16.07) | 4.34 (2.14) | 1.26 (0.59) | 165.26 (35.67) | 26.08 (3.27) | 64.90 (10.79) | 28.04 (4.21) |
| 4 | 7.92 (3.19) | 9.69 (4.22) | 71.86 (33.04) | 3.31 (2.25) | 0.95 (0.59) | 160.55 (34.17) | 26.22 (2.94) | 66.24 (11.18) | 28.06 (4.18) |
| 5 | 6.81 (3.06) | 9.22 (2.95) | 80.55 (20.33) | 2.32 (0.92) | 0.79 (0.30) | 146.19 (25.14) | 25.64 (3.25) | 70.19 (8.81) | 27.48 (4.94) |
| 6 | 8.56 (3.97) | 10.81 (3.65) | 81.91 (20.02) | 3.01 (1.53) | 0.97 (0.53) | 168.65 (32.44) | 25.99 (2.82) | 65.36 (9.60) | 27.97 (4.12) |
| 7 | 7.17 (2.99) | 8.81 (3.30) | 83.94 (14.31) | 3.21 (1.42) | 1.15 (0.53) | 160.38 (20.35) | 25.85 (3.23) | 63.78 (5.58) | 27.78 (4.70) |
| Mean (SD) | 7.7 (1.0) | 9.6 (1.0) | 77.5 (4.9) | 3.7 (1.1) | 1.1 (0.2) | 160.7 (7.1) | 26 (0.3) | 66.5 (2.2) | 27.9 (0.3) |

The mean and standard deviation of all daily time-weighted average estimates of each environmental exposure throughout the study period is shown by participant. PM_2.5_ = particulate matter < 2.5 μm (μg/m^3^); PM_10_ = particulate matter < 10 μm (μg/m^3^); CO = carbon monoxide (μg/m^3^); NO_2_ = nitrogen dioxide (μg/m^3^); SO_2_ = sulfur dioxide (μg/m^3^); O_3_ = ozone (μg/m^3^); SD = standard deviation

**Supplementary Table 3. Feasibility and Acceptability Surveys**

| **Feasibility metrics** | **Agree/Strongly Agree (N (%))** |
| --- | --- |
| I would like to use this system more often. | 3 (75) |
| I find this system to be more complicated than it should be. | 1 (25) |
| I think the system is simple and easy to use. | 3 (75) |
| I need technical support to use this system. | 1 (25) |
| I find the system functions smoothly and is well integrated. | 4 (100) |
| I think there are a lot of irregularities in the system. | 0 (0) |
| I think most people can learn this system quickly. | 3 (75) |
| I find this system to be time-consuming. | 0 (0) |
| I feel confident while using this system. | 3 (75) |
| I think there are a lot of things to learn before I can start using this system. | 2 (50) |
| **Acceptability metrics** | **N "Yes" (%)** |
| Thought wearing the Fitbit would be not at all or a little annoying. | 2 (50) |
| Comfortable or very comfortable wearing a Fitbit in front of friends. | 4 (100) |
| Thought it would take/it took a few hours or less to learn to use Fitbit. | 4 (100) |
| Thought would/did enjoy using the Fitbit a lot. | 4 (100) |
| Did not find the Fitbit to be too complicated. | 4 (100) |
| If asked to wear the Fitbit for longer, would do so. | 2 (50) |
| Thought would/did change activity a little or a lot while wearing Fitbit. | 4 (100) |
| Checked Fitbit several times per day or more for activity information. | 3 (75) |
| Checked the Fitbit app or website for activity information. | 4 (100) |
| Changed activity based on Fitbit app or website information. | 4 (100) |
| Thought would have to/had to remove the Fitbit once per day. | 4 (100) |
| Took off Fitbit for bathing. | 3 (75) |
| Took off Fitbit for sports. | 0 (0) |
| Took off Fitbit for swimming. | 2 (50) |
| Took off Fitbit for sleep. | 0 (0) |
| Took off Fitbit for Other (e.g., school). | 1 (25) |
| Sometimes forgot to put Fitbit back on after taking it off. | 3 (75) |
| I found the Fitbit too complicated. | 1 (25) |
| I felt confident using the Fitbit. | 3 (75) |

Descriptive statistics of participant responses to feasibility and acceptability surveys are shown (N = 4). Feasibility scores range from 1 (“Strongly Disagree”) to 5 (“Strongly Agree”). Acceptability metrics were posed as “Yes” or “No” questions.

**Supplementary Table 4. Pearson’s Correlation Coefficients for Bivariate Correlations**

| Variable | Anxious | Worried | Nervous | Sad | Hopeless | Tired | Happy | PM_2.5_ | PM_10_ | O_3_ | NO_2_ | SO_2_ | CO | Temp | RH | HI | Sleep_D | Sleep_S | RHR | BR | HRV_D | HRV_S | Active |
| --- | --- | --- | --- | --- | --- | --- | --- | --- | --- | --- | --- | --- | --- | --- | --- | --- | --- | --- | --- | --- | --- | --- | --- |
| Anxious | 1 |  |  |  |  |  |  |  |  |  |  |  |  |  |  |  |  |  |  |  |  |  |  |
| Worried | 0.7010 | 1 |  |  |  |  |  |  |  |  |  |  |  |  |  |  |  |  |  |  |  |  |  |
| Nervous | 0.6414 | 0.5424 | 1 |  |  |  |  |  |  |  |  |  |  |  |  |  |  |  |  |  |  |  |  |
| Sad | 0.6054 | 0.6379 | 0.5539 | 1 |  |  |  |  |  |  |  |  |  |  |  |  |  |  |  |  |  |  |  |
| Hopeless | 0.5674 | 0.6510 | 0.5752 | 0.6584 | 1 |  |  |  |  |  |  |  |  |  |  |  |  |  |  |  |  |  |  |
| Tired | 0.2695 | 0.2809 | 0.3579 | 0.1836 | 0.2332 | 1 |  |  |  |  |  |  |  |  |  |  |  |  |  |  |  |  |  |
| Happy | 0.0572 | 0.0367 | 0.1217 | -0.0236 | -0.0113 | -0.0289 | 1 |  |  |  |  |  |  |  |  |  |  |  |  |  |  |  |  |
| PM_2.5_ | -0.0935 | 0.0129 | -0.0060 | -0.0773 | -0.0123 | -0.0182 | -0.0155 | 1 |  |  |  |  |  |  |  |  |  |  |  |  |  |  |  |
| PM_10_ | -0.1364 | -0.0298 | -0.0725 | -0.1143 | -0.0724 | -0.0457 | -0.0282 | 0.9019 | 1 |  |  |  |  |  |  |  |  |  |  |  |  |  |  |
| O_3_ | 0.0794 | 0.1617 | 0.0858 | 0.1332 | 0.1147 | 0.0016 | 0.1248 | 0.4005 | 0.3561 | 1 |  |  |  |  |  |  |  |  |  |  |  |  |  |
| NO_2_ | -0.0081 | 0.0675 | 0.1198 | 0.0172 | 0.1388 | 0.0541 | -0.0669 | 0.3404 | 0.1802 | -0.4209 | 1 |  |  |  |  |  |  |  |  |  |  |  |  |
| SO_2_ | 0.0394 | 0.1391 | 0.1916 | 0.0750 | 0.2058 | 0.0794 | -0.0091 | 0.4846 | 0.3152 | 0.2464 | 0.5823 | 1 |  |  |  |  |  |  |  |  |  |  |  |
| CO | -0.0568 | -0.0104 | -0.0178 | -0.1230 | -0.0850 | 0.0501 | 0.0240 | 0.5303 | 0.4364 | 0.0550 | 0.4111 | 0.2415 | 1 |  |  |  |  |  |  |  |  |  |  |
| Temp | -0.1668 | -0.0982 | -0.0250 | -0.2047 | -0.1171 | 0.0950 | 0.0161 | 0.5504 | 0.4763 | 0.4282 | 0.0856 | 0.4508 | -0.041 | 1 |  |  |  |  |  |  |  |  |  |
| RH | 0.0129 | 0.0153 | 0.0100 | 0.0120 | -0.0074 | -0.0454 | -0.0665 | 0.1362 | 0.1190 | -0.4335 | 0.3366 | -0.0794 | 0.2532 | -0.1775 | 1 |  |  |  |  |  |  |  |  |
| HI | -0.1505 | -0.1124 | -0.0299 | -0.2088 | -0.1096 | 0.0808 | 0.0432 | 0.5635 | 0.4863 | 0.4045 | 0.1224 | 0.4740 | 0.0010 | 0.9643 | -0.0891 | 1 |  |  |  |  |  |  |  |
| Sleep_D | 0.0035 | -0.0776 | 0.0311 | -0.0483 | 0.0497 | -0.1470 | -0.0867 | 0.0796 | 0.0223 | 0.0177 | 0.0030 | 0.0064 | -0.0517 | 0.0793 | -0.0678 | 0.0952 | 1 |  |  |  |  |  |  |
| Sleep_S | 0.0339 | -0.0566 | 0.0510 | 0.0220 | 0.0000 | -0.2355 | -0.2361 | 0.0688 | 0.0552 | 0.0784 | -0.0979 | 0.0075 | -0.1126 | 0.1174 | -0.0547 | 0.1060 | 0.7398 | 1 |  |  |  |  |  |
| RHR | 0.0034 | -0.0243 | 0.1365 | 0.0073 | 0.2339 | -0.2438 | -0.2048 | 0.0770 | 0.1373 | 0.0006 | -0.0913 | -0.0729 | -0.1239 | 0.0949 | 0.1094 | 0.0913 | 0.1094 | 0.3889 | 1 |  |  |  |  |
| BR | 0.4535 | 0.4533 | 0.5455 | 0.4012 | 0.3618 | 0.2611 | 0.4082 | -0.0170 | -0.0179 | 0.1254 | 0.1093 | 0.0867 | 0.0888 | -0.0489 | 0.0786 | -0.0241 | -0.1572 | -0.2580 | -0.1221 | 1 |  |  |  |
| HRV_D | 0.2154 | 0.1284 | 0.2783 | 0.1246 | -0.0294 | 0.0111 | 0.1416 | -0.0277 | 0.0466 | 0.0349 | -0.2392 | -0.1601 | -0.0889 | -0.0814 | 0.0044 | -0.1095 | -0.0557 | -0.0359 | -0.2431 | 0.4099 | 1 |  |  |
| HRV_S | 0.2861 | 0.2557 | 0.2998 | 0.2170 | 0.1074 | -0.0472 | 0.0852 | -0.0306 | 0.0261 | 0.0270 | -0.1955 | -0.1504 | -0.0829 | -0.1860 | 0.0080 | -0.2217 | -0.0691 | 0.0080 | -0.0896 | 0.4215 | 0.9039 | 1 |  |
| Active | 0.1361 | -0.0083 | -0.1404 | 0.0685 | 0.0062 | -0.1015 | -0.0953 | 0.0012 | 0.0466 | -0.0481 | 0.0036 | -0.0608 | -0.0345 | 0.0665 | 0.0434 | 0.0656 | -0.241 | -0.1428 | 0.1863 | 0.0295 | 0.0530 | 0.0772 | 1 |

Bivariate Pearson’s correlation coefficients for each pair of continuous variables are shown. PM_2.5_ = particulate matter < 2.5 μm (μg/m^3^); PM_10_ = particulate matter < 10 μm (μg/m^3^); CO = carbon monoxide (μg/m^3^); NO_2_ = nitrogen dioxide (μg/m^3^); SO_2_ = sulfur dioxide (μg/m^3^); O_3_ = ozone (μg/m^3^); Temperature = ambient temperature (°C); Humidity = relative humidity (%); Heat index = heat index derived from Rothfusz regression (°C); Sleep_D = total hours spent sleeping within a 24 hour period, including naps; Sleep_S = representation of sleep quality ranging from 0 to 100 (optimal) based on sleep duration and time spent in REM and deep sleep phases; HRV_D = average daily root mean square of successive differences (RMSSD) of heart rate; HRV_S = RMSSD of heart rate during deep sleep phases from the longest sleep period over the past day; RHR = average daily heart rate while participant was still and well-rested; BR = average daily breaths/minute; Active = daily minutes with heart rate elevated above resting.

**Supplementary Table 5. False Discovery Rate Adjusted *P-*values for Bivariate Correlations**

| Variable | Anxious | Worried | Nervous | Sad | Hopeless | Tired | Happy | PM_2.5_ | PM_10_ | O_3_ | NO_2_ | SO_2_ | CO | Temp | RH | HI | Sleep_D | Sleep_S | RHR | BR | HRV_D | HRV_S | Active |
| --- | --- | --- | --- | --- | --- | --- | --- | --- | --- | --- | --- | --- | --- | --- | --- | --- | --- | --- | --- | --- | --- | --- | --- |
| Anxious |  |  |  |  |  |  |  |  |  |  |  |  |  |  |  |  |  |  |  |  |  |  |  |
| Worried | <.001 |  |  |  |  |  |  |  |  |  |  |  |  |  |  |  |  |  |  |  |  |  |  |
| Nervous | <.001 | <.001 |  |  |  |  |  |  |  |  |  |  |  |  |  |  |  |  |  |  |  |  |  |
| Sad | <.001 | <.001 | <.001 |  |  |  |  |  |  |  |  |  |  |  |  |  |  |  |  |  |  |  |  |
| Hopeless | <.001 | <.001 | <.001 | <.001 |  |  |  |  |  |  |  |  |  |  |  |  |  |  |  |  |  |  |  |
| Tired | <.001 | <.001 | <.001 | .05 | .01 |  |  |  |  |  |  |  |  |  |  |  |  |  |  |  |  |  |  |
| Happy | .63 | .78 | .25 | .91 | .97 | .85 |  |  |  |  |  |  |  |  |  |  |  |  |  |  |  |  |  |
| PM_2.5_ | .41 | .96 | .98 | .54 | .97 | .93 | .94 |  |  |  |  |  |  |  |  |  |  |  |  |  |  |  |  |
| PM_10_ | .17 | .86 | .56 | .34 | .59 | .71 | .85 | <.001 |  |  |  |  |  |  |  |  |  |  |  |  |  |  |  |
| O_3_ | .50 | .08 | .48 | .24 | .35 | 1.00 | .15 | <.001 | <.001 |  |  |  |  |  |  |  |  |  |  |  |  |  |  |
| NO_2_ | .98 | .56 | .30 | .94 | .24 | .64 | .52 | <.001 | .02 | <.001 |  |  |  |  |  |  |  |  |  |  |  |  |  |
| SO_2_ | .78 | .15 | .04 | .56 | .04 | .46 | .97 | <.001 | <.001 | <.001 | <.001 |  |  |  |  |  |  |  |  |  |  |  |  |
| CO | .65 | .97 | .94 | .30 | .52 | .68 | .88 | <.001 | <.001 | .60 | <.001 | <.001 |  |  |  |  |  |  |  |  |  |  |  |
| Temp | .07 | .37 | .91 | .03 | .35 | .36 | .93 | <.001 | <.001 | <.001 | .37 | <.001 | .72 |  |  |  |  |  |  |  |  |  |  |
| RH | .96 | .94 | .97 | .97 | .98 | .71 | .52 | .10 | .17 | <.001 | <.001 | .42 | <.001 | .02 |  |  |  |  |  |  |  |  |  |
| HI | .12 | .30 | .87 | .03 | .38 | .45 | .71 | <.001 | <.001 | <.001 | .15 | <.001 | 1.00 | <.001 | .35 |  |  |  |  |  |  |  |  |
| Sleep_D | .99 | .57 | .89 | .78 | .78 | .19 | .50 | .54 | .93 | .94 | .99 | .98 | .73 | .54 | .62 | .45 |  |  |  |  |  |  |  |
| Sleep_S | .86 | .71 | .76 | .93 | 1.00 | .01 | .01 | .61 | .71 | .55 | .45 | .98 | .36 | .34 | .71 | .39 | <.001 |  |  |  |  |  |  |
| RHR | .99 | .92 | .28 | .98 | .03 | .01 | .02 | .54 | .20 | 1.00 | .45 | .56 | .28 | .44 | .35 | .45 | .36 | <.001 |  |  |  |  |  |
| BR | <.001 | <.001 | <.001 | <.001 | <.001 | .01 | <.001 | .95 | .94 | .32 | .39 | .52 | .51 | .76 | .56 | .92 | .15 | .005 | .32 |  |  |  |  |
| HRV_D | .03 | .32 | .01 | .35 | .91 | .97 | .20 | .91 | .76 | .85 | .01 | .15 | .50 | .54 | .99 | .38 | .70 | .84 | .01 | <.001 |  |  |  |
| HRV_S | .002 | .01 | .002 | .05 | .46 | .76 | .51 | .88 | .91 | .91 | .05 | .18 | .53 | .07 | .98 | .02 | .59 | .98 | .48 | <.001 | <.001 |  |  |
| Active | .33 | .98 | .35 | .71 | .99 | .45 | .44 | 1.00 | .76 | .76 | .99 | .67 | .85 | .62 | .78 | .63 | .03 | .30 | .10 | .91 | .76 | .62 |  |

False discovery rate-adjusted *P*-values (Benjamini-Hochberg method) are shown for Pearson’s correlations between each pair of continuous variables. PM_2.5_ = particulate matter < 2.5 μm (μg/m^3^); PM_10_ = particulate matter < 10 μm (μg/m^3^); CO = carbon monoxide (μg/m^3^); NO_2_ = nitrogen dioxide (μg/m^3^); SO_2_ = sulfur dioxide (μg/m^3^); O_3_ = ozone (μg/m^3^); Temperature = ambient temperature (°C); Humidity = relative humidity (%); Heat index = heat index derived from Rothfusz regression (°C); Sleep_D = total hours spent sleeping within a 24 hour period, including naps; Sleep_S = representation of sleep quality ranging from 0 to 100 (optimal) based on sleep duration and time spent in REM and deep sleep phases; HRV_D = average daily root mean square of successive differences (RMSSD) of heart rate; HRV_S = RMSSD of heart rate during deep sleep phases from the longest sleep period over the past day; RHR = average daily heart rate while participant was still and well-rested; BR = average daily breaths/minute; Active = daily minutes with heart rate elevated above resting.

**Supplementary Table 6. Sensitivity Indices for Bivariate Correlations**

| Variable | Anxious | Worried | Nervous | Sad | Hopeless | Tired | Happy | PM2.5 | PM10 | O_3_ | NO_2_ | SO_2_ | CO | Temp | RH | HI | Sleep_D | Sleep_S | RHR | BR | HRV_D | HRV_S | Active |
| --- | --- | --- | --- | --- | --- | --- | --- | --- | --- | --- | --- | --- | --- | --- | --- | --- | --- | --- | --- | --- | --- | --- | --- |
| Anxious |  |  |  |  |  |  |  |  |  |  |  |  |  |  |  |  |  |  |  |  |  |  |  |
| Worried | 0.0360 |  |  |  |  |  |  |  |  |  |  |  |  |  |  |  |  |  |  |  |  |  |  |
| Nervous | 0.0357 | 0.0541 |  |  |  |  |  |  |  |  |  |  |  |  |  |  |  |  |  |  |  |  |  |
| Sad | 0.0342 | 0.0329 | 0.0458 |  |  |  |  |  |  |  |  |  |  |  |  |  |  |  |  |  |  |  |  |
| Hopeless | 0.0505 | 0.0338 | 0.0479 | 0.0327 |  |  |  |  |  |  |  |  |  |  |  |  |  |  |  |  |  |  |  |
| Tired | 0.0367 | 0.0525 | 0.0383 | 0.0637 | 0.0484 |  |  |  |  |  |  |  |  |  |  |  |  |  |  |  |  |  |  |
| Happy | 0.0554 | 0.0573 | 0.0401 | 0.0554 | 0.0702 | 0.0464 |  |  |  |  |  |  |  |  |  |  |  |  |  |  |  |  |  |
| PM2.5 | 0.0185 | 0.0225 | 0.0259 | 0.0383 | 0.0443 | 0.0223 | 0.0242 |  |  |  |  |  |  |  |  |  |  |  |  |  |  |  |  |
| PM10 | 0.0201 | 0.0277 | 0.0264 | 0.0316 | 0.0379 | 0.0230 | 0.0270 | 0.0040 |  |  |  |  |  |  |  |  |  |  |  |  |  |  |  |
| O_3_ | 0.0258 | 0.0187 | 0.0363 | 0.0326 | 0.0363 | 0.0190 | 0.0293 | 0.0171 | 0.0167 |  |  |  |  |  |  |  |  |  |  |  |  |  |  |
| NO_2_ | 0.0318 | 0.0355 | 0.0355 | 0.0519 | 0.0617 | 0.0256 | 0.0434 | 0.0239 | 0.0303 | 0.0091 |  |  |  |  |  |  |  |  |  |  |  |  |  |
| SO_2_ | 0.0289 | 0.0404 | 0.0332 | 0.0503 | 0.0639 | 0.0265 | 0.0546 | 0.0249 | 0.0276 | 0.0100 | 0.0059 |  |  |  |  |  |  |  |  |  |  |  |  |
| CO | 0.0257 | 0.0196 | 0.0342 | 0.0405 | 0.0462 | 0.0234 | 0.0193 | 0.0217 | 0.0272 | 0.0119 | 0.0174 | 0.0215 |  |  |  |  |  |  |  |  |  |  |  |
| Temp | 0.0288 | 0.0319 | 0.0205 | 0.0349 | 0.0532 | 0.0231 | 0.0320 | 0.0181 | 0.0228 | 0.0191 | 0.0228 | 0.0167 | 0.0314 |  |  |  |  |  |  |  |  |  |  |
| RH | 0.0304 | 0.0364 | 0.0279 | 0.0293 | 0.0284 | 0.0182 | 0.0228 | 0.0301 | 0.0233 | 0.0238 | 0.0196 | 0.0194 | 0.0242 | 0.0268 |  |  |  |  |  |  |  |  |  |
| HI | 0.0286 | 0.0328 | 0.0256 | 0.0335 | 0.0510 | 0.0254 | 0.0314 | 0.0158 | 0.0204 | 0.0220 | 0.0226 | 0.0206 | 0.0254 | 0.0010 | 0.0283 |  |  |  |  |  |  |  |  |
| Sleep_D | 0.0334 | 0.0298 | 0.0413 | 0.0282 | 0.0489 | 0.0288 | 0.0193 | 0.0130 | 0.0081 | 0.0391 | 0.0468 | 0.0314 | 0.0389 | 0.0216 | 0.0397 | 0.0176 |  |  |  |  |  |  |  |
| Sleep_S | 0.0377 | 0.0263 | 0.0472 | 0.0357 | 0.0544 | 0.0525 | 0.0504 | 0.0200 | 0.0242 | 0.0428 | 0.0551 | 0.0394 | 0.0469 | 0.0206 | 0.0244 | 0.0126 | 0.0256 |  |  |  |  |  |  |
| RHR | 0.0470 | 0.0685 | 0.0558 | 0.0439 | 0.0821 | 0.0702 | 0.1108 | 0.0190 | 0.0343 | 0.0375 | 0.0862 | 0.0822 | 0.0440 | 0.0302 | 0.0334 | 0.0297 | 0.0176 | 0.0550 |  |  |  |  |  |
| BR | 0.0908 | 0.0810 | 0.0600 | 0.1086 | 0.1188 | 0.0997 | 0.0609 | 0.0148 | 0.0228 | 0.0226 | 0.0569 | 0.0447 | 0.0362 | 0.0215 | 0.0355 | 0.0229 | 0.0296 | 0.0865 | 0.1398 |  |  |  |  |
| HRV_D | 0.0832 | 0.0802 | 0.0928 | 0.1027 | 0.0922 | 0.0487 | 0.0465 | 0.0196 | 0.0304 | 0.0285 | 0.0377 | 0.0581 | 0.0144 | 0.0429 | 0.0667 | 0.0423 | 0.0202 | 0.0355 | 0.1044 | 0.0603 |  |  |  |
| HRV_S | 0.0674 | 0.0499 | 0.0723 | 0.0866 | 0.0541 | 0.0315 | 0.0436 | 0.0200 | 0.0192 | 0.0368 | 0.0276 | 0.0493 | 0.0252 | 0.0485 | 0.0618 | 0.0494 | 0.0212 | 0.0285 | 0.0717 | 0.0742 | 0.0284 |  |  |
| Active | 0.0549 | 0.0422 | 0.0760 | 0.0438 | 0.0549 | 0.0362 | 0.0614 | 0.0386 | 0.0201 | 0.0203 | 0.0569 | 0.0228 | 0.0240 | 0.0201 | 0.0121 | 0.0288 | 0.0253 | 0.0549 | 0.0733 | 0.1178 | 0.1423 | 0.1362 |  |

Sensitivity indices are shown for Pearson’s correlations between each pair of continuous variables, calculated as the mean absolute value of the change in correlation coefficient after sequentially excluding single participants. PM_2.5_ = particulate matter < 2.5 μm (μg/m^3^); PM_10_ = particulate matter < 10 μm (μg/m^3^); CO = carbon monoxide (μg/m^3^); NO_2_ = nitrogen dioxide (μg/m^3^); SO_2_ = sulfur dioxide (μg/m^3^); O_3_ = ozone (μg/m^3^); Temperature = ambient temperature (°C); Humidity = relative humidity (%); Heat index = heat index derived from Rothfusz regression (°C); Sleep_D = total hours spent sleeping within a 24 hour period, including naps; Sleep_S = representation of sleep quality ranging from 0 to 100 (optimal) based on sleep duration and time spent in REM and deep sleep phases; HRV_D = average daily root mean square of successive differences (RMSSD) of heart rate; HRV_S = RMSSD of heart rate during deep sleep phases from the longest sleep period over the past day; RHR = average daily heart rate while participant was still and well-rested; BR = average daily breaths/minute; Active = daily minutes with heart rate elevated above resting.
